# Cone-Beam Computed Tomographic Evaluation of Root Canal Morphology of Maxillary Premolars in a Saudi Population

**DOI:** 10.1155/2018/8170620

**Published:** 2018-08-15

**Authors:** Abdullah Alqedairi, Hussam Alfawaz, Yousef Al-Dahman, Faisal Alnassar, Asma Al-Jebaly, Sara Alsubait

**Affiliations:** ^1^Division of Endodontics, Department of Restorative Dental Sciences, College of Dentistry, King Saud University, Riyadh, Saudi Arabia; ^2^Endodontist, Ministry of Health, Saudi Arabia; ^3^Endodontic Division, Restorative Department, Majmaah University, Saudi Arabia; ^4^General Practitioner, Riyadh, Saudi Arabia

## Abstract

**Objective:**

The aim of the study was to investigate the root canal morphology of maxillary first and second premolars in a Saudi population using Cone-Beam Computed Tomography (CBCT).

**Methods:**

This retrospective cross-sectional study assessed CBCT images of 707 Saudi patients. The number of roots and canal configuration were identified based on Vertucci's classification. Fisher's exact Chi-square tests were performed to analyze the association between sex and number of roots and sex and root canal configuration.

**Results:**

Most teeth had two roots in maxillary first premolars (75.1%) and one root in maxillary second premolars (85.2%). Type IV was the most prevalent canal configuration in maxillary first premolars (69.1%), while Type I was the most in maxillary second premolars (49.4%). All types of canal configurations were observed in maxillary premolars except Type VII for the maxillary second premolar. Chi-square tests showed no significant association between gender and number of roots and sex and root canal configuration in both maxillary first and second premolars although higher number of roots was seen in men (P > 0.05).

**Conclusion:**

Most maxillary first premolars had two roots with Type IV being the most predominant canal configuration, while a single root with Type I canal configuration was the most frequently observed morphology in maxillary second premolars. In maxillary first premolars, 21.3% had one canal apically, 75.4% had two canals apically, and 3.3% had three canals apically. In maxillary second premolars, 80.2% had one canal apically, 18.9% had two canals apically, and 0.9% had three canals apically.

## 1. Introduction

Proper knowledge of the anatomy of the root canal system and its morphological variations plays a significant role in all the steps of endodontic treatment [1–3]. Therefore, the clinician should have a thorough understanding of the detailed anatomy of the root canal in order to utilize the most appropriate treatment techniques and protocols and thereby increase the success rate [4].

The anatomical variations of the root canal system are crucial in endodontic treatment. The untreated missing root canal will lead to persistent presence of microorganisms and necrotic tissue inside the canal, which may result in development of apical pathosis [5].

Different classifications have described the root canal systems of human permanent teeth including the Weine [6], Vertucci [1], and Gulabivala [7] classifications. Vertucci's classification is considered the most widely used and includes eight categories: Type I (1), Type II (2-1), Type III (1-2-1), Type IV (2), Type V (1-2), Type VI (2-1-2), Type VII (1-2-1-2), and Type VIII (3). Root canal treatment of premolar teeth is reportedly very challenging due to anatomical variations in the number of roots and types of canal configurations [3, 8–11].

The maxillary first premolars are considered among the most difficult teeth to be endodontically treated because of the number of roots and canals, the direction and longitudinal depressions of the roots, the various pulp cavity configurations, and the difficulties in visualizing the apical limit on periapical radiographs [12]. Moreover, high variability has been reported in the root canal morphology of the maxillary second premolars [1, 13–15].

Different methods have been utilized to investigate root canal anatomy including* in vivo* and* in vitro* methods. The* in vivo* techniques include clinical evaluation during root canal treatment, retrospective assessment of patient records, conventional radiographic evaluation, and advanced radiographic techniques such as cone-beam computed radiography (CBCT) [16–18], while the* in vitro* methods include canal staining and tooth clearing [1, 19], root sectioning [6], microscopic examination, examination of conventional radiographs, and using three-dimensional modalities such as microcomputed tomography (*μ*-CT) [20–22].

The CBCT technique has the ability to detect root canal morphology as precisely as the root canal staining and clearing techniques which, in the past, were considered superior to conventional techniques used for studying the root canal system because of their ability to provide three-dimensional views and complete morphologic details [5].

To the best of our knowledge, no study has so far evaluated the root canal morphology of maxillary premolars of both sexes in a Saudi population using CBCT. Therefore, the aim of the study was to investigate the root canal morphology of maxillary first and second premolars in a Saudi population using CBCT.

## 2. Materials and Methods

Seven hundred and seven CBCT images of Saudi patients (396 female, 311 male) aged between 16 and 71 years, with average age of 41.5 years, seeking routine dental treatment who were referred to the Radiology Department of the College of Dentistry, King Saud University, between 2015 and 2017 were collected.

The sampling was purposive where the presence of at least one maxillary first and/or second premolar with fully developed roots was the inclusion criterion. Unclear or distorted CBCT images, previously endodontically initiated or treated teeth, teeth with posts or crowns, periapical lesions, and any physiological or pathological process such as immature apex were excluded. The total final sample, consisting of 334 maxillary first premolars and 318 maxillary second premolars, was evaluated in terms of number of roots and root canal configuration. The data were observed and recorded for the number of roots and canal configuration based on Vertucci's classification [1]. The sex of the patients was also recorded.

The CBCT images were accessed and evaluated at the Radiology Department of the College of Dentistry, King Saud University, by two endodontists for the number of roots and root canal configuration using the using the Planmeca Romexis Viewer software (Planmeca, Roselle IL). The images were collected from different CBCT machines: CS9300 3D digital imaging system (Carestream, Rochester, NY) with a voxel size of 90 to 300 *μ*m and Planmeca ProMax 3D (Planmeca, Roselle IL) with a voxel size of ≤ 200 *μ*m.

To ensure the reliability of the results, inter- and intraexaminer reliabilities were measured by identifying the root canal anatomy of maxillary premolars of 30 randomly selected CBCT images according to the evaluation criteria. For intraexaminer reliability, the same images were evaluated after 1 week. Both inter- and intraexaminer reliabilities were calculated using the interclass correlation coefficient (ICC). Data were analyzed with Fisher's exact test using SPSS software version 22 (SPSS Inc., Chicago, IL, USA). The significance was set at the 95% confidence level.

## 3. Results

For interexaminer reliability, the ICC was 0.886 (excellent) for the number of roots and 0.625 (good) for canal configuration. For intraexaminer reliability, the ICC was 1 for the first examiner with regard to number of roots and canal configuration and 1 and 0.95 for the second examiner with regard to number of roots and canal configuration, respectively. The ICCs verified that the procedure was reliable for the evaluations and measurements performed by the two observers.

**Figure 1:**
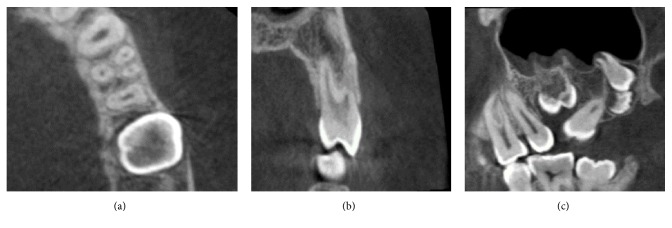
CBCT of maxillary first premolar with three roots: (a) the axial plane, (b) the coronal plane, and (c) the sagittal plane.

The number of roots recorded in maxillary first and second premolars was up to three roots (Figure 1). In maxillary first premolars, most teeth had two roots (75.1%), while in maxillary second premolars most were single rooted (85.2%). Type IV canal configuration (69.1%) was the most prevalent observation in maxillary first premolars, while Type I (49.4%) was the most prevalent configuration in maxillary second premolars. The frequency and percentage of number of roots and canal configuration in maxillary first and second premolars are shown in Tables 1 and 2, respectively.

A higher percentage of three-rooted maxillary first premolars was observed in men (2.3%) and none in women (0%), while the most predominant root morphology seen in both sexes was two rooted, with no statistically significant difference (P = 0.078). In canal configurations, there were no statistically significant differences in all types between the sexes (P = 0.095).

In maxillary second premolars, there was no statistically significant difference in number of roots between the sexes (P = 0.383). Although Type III, VI, and VIII canal configurations were more frequent in female patients and Type II, IV, and V in male patients, the difference was not statistically significant (P = 0.257).

Both right and left maxillary first premolars were present in 170 patients. Symmetrical number of roots and canal configuration were seen in 91.2% of teeth, while 2.3% showed symmetrical number of roots but different canal configuration and 0.6% showed symmetrical canal configuration but different number of roots. Moreover, 5.9% of teeth did not show any type of symmetry.

In 163 patients, right and left maxillary second premolars were present. Symmetrical numbers of roots and canal configuration were seen in 85.3% of teeth, while 11% showed symmetrical number of roots but different canal configuration. Only 3.7% showed no type of symmetry.

## 4. Discussion

Patient ethnicity is an incontrovertible factor that may affect the perception of the clinician for the suspected root canal anatomy. In the present study, maxillary premolars in a Saudi population, where the majority is Arab, presented with up to three roots and included most of Vertucci's types of canal configuration with similarities and differences to the other reported studies in different populations. Moreover, sex predilection and symmetrical anatomy were investigated to evaluate their significance in the prediction of root canal anatomy.

CBCT is reportedly an excellent tool for more accurately detecting root canal anatomy than intraoral periapical radiography due to its ability to evaluate and assess root canal morphology in three dimensions [5, 23–27]. The data of this retrospective study were collected from a single center, King Saud University Dental Hospital, which is considered one of the largest governmental dental institutes providing free dental services to a large portion of the Saudi population from different regions. To avoid exposing a large number of patients to unnecessary radiation, a CBCT imaging database was accessed regardless of the voxel size to achieve a larger sample size.

In maxillary first premolars, the prevalence of one root was reported to be 22% to 66%, of two roots 33% to 84%, and of three roots 0% to 6% [28–34]. In maxillary second premolars, the prevalence of one root was reported to be 69.6% to 90.3%, of two roots 9.7% to 29.7%, and of three roots 0% to 1.6% [30, 34–37].

In maxillary first premolars in the present study, the most frequently observed root morphology was two rooted (75.1%), followed by single rooted (23.7%), and three rooted (1.2%). These findings are consistent with those reported by Elkady and Allouba, using CBCT, who reported that, in maxillary first premolars in Saudi subpopulation, the prevalence of one root was 28.3% and of two roots 71.7%, while no three roots were observed [38]. In another study with a Saudi population, using visual, digital radiography and transverse sectioning methods, in maxillary first premolars, the prevalence of one root was reported to be 17.9%, of two roots 80.9%, and of three roots 1.2% [16].

Regarding canal configuration, all types of Vertucci's classification were observed in this study, which it is in line with Vertucci and Gegauff's findings [32]. Most patients had Type IV canal configurations, followed by Type I, Type II, and finally Type VII. In comparison to other studies included in a literature review [39], Type IV canal configuration was reported to be 65.3% in Saudi population, followed by Type II (19.7%), Type I (7.6%), Type III (3.3%), Types V, VI, and VII (3.3%), and finally Type VIII (0.8%).

In maxillary second premolars, up to three-rooted teeth were observed. The highest prevalence was single rooted followed by double rooted and three rooted (0.3%). Additionally, the majority exhibited Type I canal configuration, followed by Type II and Type IV. These findings coincide with those of other studies conducted in Saudi Arabia, where one root was observed in 76.4% and 67%, two roots in 23.6% and 30%, and three roots in 0% and 3% teeth [38, 40]. Moreover, Type I canal configuration was seen in 36.3% and 17%, Type II in 10.9% and 7%, and Type IV in 23.6% and 23% teeth [38, 40].

Compared to a recent CBCT study by Abella et al. [37] in Spanish population, in maxillary first premolars, the most prevalent root morphology observed was two rooted (51.4%) and most of the teeth exhibited Type IV canal configuration (52.8%), while in maxillary second premolars, the most prevalent root morphology seen was single rooted (82.9%) and most of these teeth exhibited Type I canal configuration (47.2%). Moreover, two-rooted maxillary first premolars were reported to be 33% with 51% having Type IV canal configuration in a Chinese population [41], 68.6% with 68% having Type I canal configuration in a Pakistani population [42], and 44.8% with 76.8% having Type IV canal configuration in a Turkish Cypriot population [43]. Additionally, single-rooted maxillary second premolars were reported to be 84% with 53.4% having Type I canal configuration in a Pakistani population [42], and Type I canal configuration was the most commonly observed in a Turkish Cypriot population (49.4%) [43].

Sex predilection regarding the number of roots and root canal morphology has been reported [44–47]. Martins et al. [48] reported that female patients in a Portuguese population had a lower number of roots in both maxillary first and second premolars with a statistically significant difference in the maxillary first premolars. Moreover, Type IV canal configuration in maxillary first premolars was more frequent in male patients and Type I canal configuration in maxillary second premolars was more in female patients with a statistical significant difference (P < 0.05), while Abella et al. [37] reported that there was no statistically significant correlation between the number of roots and the sexes in Spanish population. In this study, there was no statistically significant correlation between sex and number of roots or sex and root canal configuration in both maxillary first and second premolars although a higher number of roots were seen in men (P > 0.05).

The degree of bilateral symmetry in root canal morphology using CBCT has been reported in different studies. For maxillary first premolars, bilateral symmetry was observed in 88.5% of teeth for the number of roots and in 77% for canal configuration in Saudi patients [38], and symmetry of 64% was reported for both number of roots and canal configuration in a Chinese population [41]. In maxillary second premolars, bilateral symmetry was observed in 84% of teeth for the number of roots and in 76% for canal configuration [38]. The findings of the present study are in agreement with those of the previous studies where a high degree of symmetry in the number of roots and canal configuration was seen in both maxillary first and second premolars.

## 5. Conclusion

Within its limitations, in this study with a Saudi population, most maxillary first premolars had two roots with Type IV being the most predominant canal configuration, while a single root with Type I canal configuration was the most frequently observed morphology in maxillary second premolars. Moreover, more than one root with different canal configurations was detected in some cases. Further studies with larger sample sizes are recommended for the generalization of our results.

## Figures and Tables

**Table 1:** The frequency and percentage of number of roots and canal configuration in maxillary first premolar teeth.

	**Number of Roots**									
		**One Root**		**Two Roots**		**Three Roots**			**Total**	
**Frequency (**%**)**	**Women**	43 (26.5%)		119 (73.5%)		0 (0%)			162 (100%)	
	**Men**	36 (20.9%)		132 (76.8%)		4 (2.3%)			172 (100%)	
	**Total**	79 (23.7%)		251 (75.1%)		4 (1.2%)			334 (100%)	

**Canal Configuration**										

		**Type I (1)**	**Type II (2-1)**	**Type III (1-2-1)**	**Type IV (2)**	**Type V (1-2)**	**Type VI (2-1-2)**	**Type VII (1-2-1-2)**	**Type VIII (3)**	**Total**

**Frequency (**%**)**	**Women**	15 (9.3%)	17 (10.5%)	5 (3.1%)	112 (69.1%)	9 (5.6%)	3 (1.8%)	0 (0%)	1 (0.6%)	162 (100%)
	**Men**	21 (12.2%)	11 (6.4%)	1 (0.6%)	124 (72.1%)	4 (2.3%)	4 (2.3%)	1 (0.6%)	6 (3.5%)	172 (100%)
	**Total**	36 (10.8%)	28 (8.4%)	6 (1.8%)	236 (70.6%)	13 (3.9%)	7 (2.1%)	1 (0.3%)	7 (2.1%)	334 (100%)

**Table 2:** The frequency and percentage of number of roots and canal configuration in maxillary second premolar teeth.

		**Number of Roots**								
			**One Root**		**Two Roots**		**Three Roots**		**Total**	
**Frequency (**%**)**		**Women**	138 (87.3%)		20 (12.7%)		0 (0%)		158 (100%)	
		**Men**	133 (83.1%)		26 (16.3%)		1 (0.6%)		159 (100%)	
		**Total**	271 (85.2%)		46 (14.5%)		1 (0.3%)		318 (100%)	

**Canal Configuration**										

		**Type I (1)**	**Type II (2-1)**	**Type III (1-2-1)**	**Type IV (2)**	**Type V (1-2)**	**Type VI (2-1-2)**	**Type VII (1-2-1-2)**	**Type VIII (3)**	**Total**

**Frequency (**%**)**	**Women**	80 (50.6%)	38 (24.0%)	10 (6.3%)	15 (9.5%)	8 (5.1%)	5 (3.2%)	0 (0%)	2 (1.3%)	158 (100%)
	**Men**	77 (48.1%)	44 (27.5%)	6 (3.8%)	22 (13.8%)	10 (6.2%)	0 (0%)	0 (0%)	1 (0.6%)	160 (100%)
	**Total**	157 (49.4%)	82 (25.8%)	16 (5%)	37 (11.6%)	18 (5.7%)	5 (1.6%)	0 (0%)	3 (0.9%)	318 (100%)

## Data Availability

The CBCT images data used to support the findings of this study are restricted by the local institutional review board at King Saud University in order to protect patients' privacy.
